# Experimental effects of brief, single bouts of walking and meditation on mood profile in young adults

**DOI:** 10.15171/hpp.2018.23

**Published:** 2018-07-07

**Authors:** Meghan K. Edwards, Paul D. Loprinzi

**Affiliations:** Physical Activity Epidemiology Laboratory, Exercise Psychology Laboratory, Department of Health, Exercise Science and Recreation Management, The University of Mississippi, University, MS 38677, USA

**Keywords:** Affect, Emotions, Exercise, psychology, Meditation, Mood, Physical activity

## Abstract

**Background: ** To examine the effects of an acute bout of aerobic exercise and meditation on mood state among young adults.

**Methods:** Participants (N= 66, mean age = 21.3 years) were randomly assigned to walk,meditate, or sit (control) for 10 minutes. Participants’ mood state was monitored before and after the intervention using the Profile of Mood States (POMS) questionnaire.

**Results: ** Significant group x time interaction effects were observed for the POMS composite scores (P=0.05). When evaluating three POMS sub scales separately (depression/dejection,anger/hostility, and fatigue/inertia), only fatigue/inertia was found to have a significant group x time effect (P=0.04). Post hoc paired t tests revealed that fatigue/inertia sub scale scores significantly decreased from baseline to post-intervention in both the exercise (P=0.03) and meditation (P<0.001) groups. However, POMS composite scores decreased significantly in the meditation group (P<0.001) but not in the exercise group (P=0.10).

**Conclusion: ** A 10-minute bout of brisk walking and meditation both improved mood state,when compared to an inactive control group. A single bout of brisk walking or meditation may offer suitable strategies to improve mood state among young adults.

## Introduction

Mood-related disorders, such as depression, pose an enormous public health burden. Psychotherapy (e.g., cognitive behavioral therapy, psychoanalysis) and pharmacotherapy are two traditional forms of intervention used to treat psychological disorders^1^ (including subclinical symptomology),^2^ with these often being implemented in conjunction.^3^ Of interest to the present study, there is ample evidence to suggest that exercise and meditation have mood-enhancing benefits and may serve as effective strategies to attenuate risk for, or symptoms associated with, psychological disorders.^4-8^ While numerous studies have demonstrated beneficial psychological effects of chronic exercise^9,10^ and meditation,^11,12^ the optimal type, intensity, frequency, and duration for these modalities are unclear.^13,14^

A number of studies have compared the effectiveness of exercise interventions with mindfulness-based interventions on improving a variety of health outcomes. For example, a 2010 review conducted by Ross and Thomas^15^ found yoga, an active mind-body practice involving a strong meditative component, to be equal or superior to exercise in nearly all assessed outcomes (with the exception of those related to physical fitness levels). Berger and Owen^16^ have presented evidence that yoga has similar (or better among males, compared to females) mood-enhancing benefits for college students compared to aerobic exercise, arguing that an aerobic-component of exercise may not be necessary for mood-enhancement. Goldin and colleagues^17^ conducted a randomized controlled trial to assess the effects of 8-weeks of aerobic exercise and mindfulness-based stress reduction (MSBR) on self-views among individuals with social anxiety disorder, finding both treatments to similarly increase positive self-views, and MSBR to reduce negative self-views to a greater extent. Multiple components make up MSBR, namely body scanning, seated meditation, yoga, and walking meditation. Few studies have made direct comparisons between exercise and mindfulness meditation as a solo intervention technique. One such study was a 16-week intervention conducted among heavy alcohol drinkers^18^; this study compared running and a mantra-based concentrative meditation technique, observing that both reduced average ethanol consumption, with exercise resulting in greater reductions. The authors highlighted a greater variability in compliance rates among the meditation group compared to the running group, and found that ethanol consumption was reduced to a greater extent among those who were more compliant with the meditations. In addition to few studies directly comparing aerobic exercise to meditation, this side-by-side comparison incorporating a psychological-based outcome (as opposed to alcohol drinking) has yet to be investigated.

In addition to longer-duration interventions, previous studies have demonstrated the ability of a single bout of exercise^19,20^ or meditation to positively influence mood state. Though comparison studies of acute exercise and mindfulness-related practices (e.g., aerobic exercise vs. yoga)^21^ have been conducted, again, there is limited evidence directly comparing the psychological effects of a single bout of exercise with a single bout of non-active meditation. The purpose of the present investigation, therefore, was to experimentally examine the impact of a single brisk walking bout and a single mindfulness meditation session on mood profile among a sample of young adults.

This study offers an important contribution to a gap in the acute exercise/meditation literature by (1) presenting a side-by-side comparison of the brisk walking and non-active meditation, and (2) utilizing an exercise/meditation protocol that would be easy to implement in a wide variety of populations and settings. Ultimately, this study sought to answer the question, “If I have 10-minutes of free time and want to engage in an activity that will benefit my mood state, should I go on a brisk walk, or meditate?”

## Materials and Methods

### Study design and participants

In brief, participants completed several baseline assessments (surveys detailed herein to follow). Following baseline measurements, participants were randomized to a walking, meditation group, or control group, with all interventions lasting for 10 minutes. Following the walk, meditation or sitting, participants’ mood was re-assessed. Participants were excluded if they: (1) were not within the target age range (18-53 years) or (2) exercised within 5 hours, consumed caffeine within 3 hours, or ate within 1 hour of coming into the laboratory.

### Walk and meditation protocol

As noted, participants were randomized into a walking (n = 22),^22^ meditation (n = 22), or control (n = 22) group. This sample size was based on previous work on this topic.^22^ Randomization occurred via computer-generated algorithm. The walking group self-selected a brisk walking pace. Specifically, participants were asked to “select a pace you would walk at if you were running late for class, a meeting, or to trying to catch the bus,” and to choose a speed they felt they could maintain for the duration of the walk. Those in the meditation group engaged in a 10-minute guided mindfulness meditation; mindfulness meditation is commonly defined as non-judgmental attention to experiences in the present moment.^23^ The implemented guided meditation cues focused on breath/body present-moment awareness (through deep breathing exercises and a full-body scan), limiting mind-wandering/letting go of distractions or worries (i.e., enhanced attentional control), and cultivating relaxation. A Yoga Alliance 200-hour registered yoga teacher (author MKE) conducted the meditations. Participants either reclined in a seated computer chair with both feet propped up on a bench or laid down on a provided yoga mat. It was important to give participants this option (sit vs. lie down), as we wanted them to feel as comfortable as possible during the meditation. All participants had their eyes closed during the meditation. Participants randomized into the control group were asked to sit in a computer chair within the laboratory for 10 minutes. They were asked to relax and sit quietly with their eyes open.

During all protocols, heart rate was assessed via a Polar heart rate monitor. We report heart rate at 4 time points: baseline, 5-minute into the walk/meditation (mid-intervention), 9-minute into the walk/meditation (end-intervention), and 3-minute post-walk/meditation (final). Notably, the assessments of heart rate during the meditation were made discretely. The researcher conducting the meditation was positioned close enough to the participant to be able to see (without having to get up and move) the Polar wrist watch displaying participants’ heart rate, thus minimizing any potential for distraction from the meditation.

### Assessment of Mood State

The Profile of Moods States (POMS) questionnaire was used to assess mood state, with the *depression-dejection* (13 items), *anger-hostility* (11 items), and *fatigue-inertia* (7 items) subscales used for the present study. In addition to evaluating each of these subscales, a POMS composite score was created by summing the responses from the *depression-dejection*,* anger-hostility* and *fatigue-inertia* subscales, with higher values indicating worse mood state.

The POMS survey has demonstrated adequate levels of internal consistency (α = 0.78-0.93)^24^ as well as criterion validity^25^ and construct validity.^26^ In our sample, internal consistency (as measured by Cronbach’s alpha) was 0.84, 0.89, and 0.76 for baseline *depression/dejection* (13 items) among the walking, meditating, and control groups (respectively). Internal consistency was 0.83, 0.71, and 0.76 for baseline *anger/hostility* (11 items) among the walking, meditating, and control groups (respectively). Internal consistency was 0.84, 0.94, and 0.86 for baseline *fatigue/inertia* (7 items) among the walking, meditating, and control groups (respectively).

### Additional assessments

As displayed in Table 1, several surveys were implemented to assess baseline demographic, behavioral and psychological characteristics across the 3 groups. Briefly, the assessed parameters included self-reported physical activity (via the International Physical Activity Questionnaire- Short Form [IPAQ-SF]),^27^ trait mindfulness (via the Freiberg Mindfulness Inventory [FMI]),^28^ typical responses to stressful situations (via the Responses to Stressful Experiences Scale [RSES]),^29^ emotion regulation (via the Difficulties in Emotion Regulation Scale [DERS]),^30^ executive functioning abilities (via the Dysexecutive Questionnaire [DEX])^31^ as well as the participants’ aversion to treadmill-based exercise/meditation and their exercise/meditation experiences and attitudes. Height was assessed using a standard stadiometer and weight was assessed using a standard scale; height and weight were used to calculate body mass index (BMI).

### Statistical analysis

Analysis was computed using SPSS software (version 24.0) and Stata software (version 12.1). Demographic differences between the 3 groups at baseline were compared via ANOVA tests of between-subject effects for continuous variables (e.g., age, BMI, physical activity, mindfulness, emotion regulation) and via chi-square tests for any nominal data (e.g., gender, race/ethnicity). For each split-plot analysis, condition (i.e., walking, meditating, or sitting) served as the between-subjects variable whereas time (i.e., baseline & post-intervention) served as the within-subjects variable. Partial eta squared (η^2^)effect size estimates were calculated for all repeated-measures analyses. Statistical significance was established as a nominal alpha = 0.05.

## Results

### Sample characteristics

Descriptive characteristics of the study sample are displayed in Table 1. The included sample sizes of the 3 groups were n = 22 in the walking group, n = 22 in the meditation group, and n = 22 in the control group. The mean (SD) ages of the groups were 21.5 (2.23) years, 22.6 (3.2) years, and 20.9 (2.5) years, respectively. Males constituted 27%, 23%, and 36% of the samples (respectively). Demographic comparisons between the 3 groups revealed that there were no statistically significant differences among the groups with regards to age (mean age = 21.4 years), gender (38.1% male), race/ethnicity (69.8% non-Hispanic white), BMI (mean BMI = 24.7 kg/m^2^), physical activity level (mean moderate-to-vigorous physical activity = 333.7 min/wk), trait mindfulness, typical responses to stressful situations, emotion regulation, executive function abilities, time since previous meal, exercise enjoyment, or meditation experience.

### Manipulation check

After completing the meditation, participants were provided (via survey assessment) a definition of mindfulness (“Mindfulness is often referred to as a mental state characterized by full attention to internal and external experiences as they occur in the present moment. Mindfulness is additionally characterized by non-judgment of, and openness to current experiences.”). Based on this definition, participants were asked to rate the extent to which they felt they (1) had practiced mindfulness, (2) felt connected to their meditation experience, (3) experienced moments of ‘inner peace’, and (4) were able to return to the meditation experienced if they noticed mind-wandering. Response options ranged from 1 (“not at all”) to 4 (“completely”). The average response (1-4) for all four questions was 3.16 (SD = 0.44). For each of the four items, no participants responded with a score of 1. The majority of responses for each of the four questions were 3’s or 4’s (96%, 83%, 78%, and 96%, respectively). As such, we feel confident that our meditation elicited the intended effects.

Additionally, at the end of the visit, participants were asked to rate the extent to which they enjoyed their meditation or exercise bout. The statements read, “How much did you enjoy your exercise bout?” and “How much did you enjoy your meditation bout?” Response options ranged from 1 “not at all” to 5 “very much.” All participants rated exercise as 3 or higher, with 68% of participants rating 4 or 5. For meditation, 91% of participants rated 4 or 5; one participant responded with 1 and one participant responded with 2, indicating that these 2 did not enjoy their meditation experiences.

### Main outcomes

**Figure 1:**
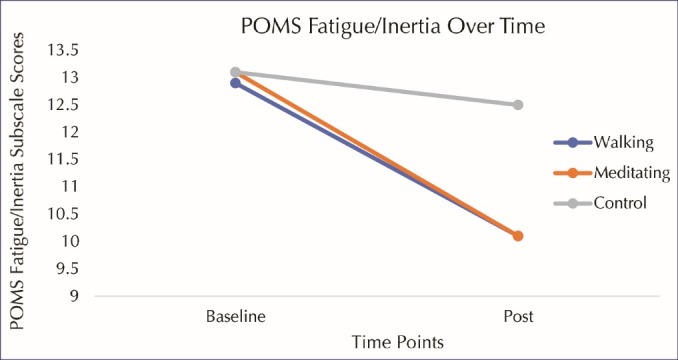
POMS (Profile of Mood States) fatigue/inertia over time. Fatigue/inertia scores significantly decreased from baseline to post-intervention in both the exercise (*P *= 0.03) and meditation (*P *< 0.001) groups. Post-intervention scores were significantly lower in the 2 intervention groups compared to the control group (*P *< 0.05).

**Figure 2:**
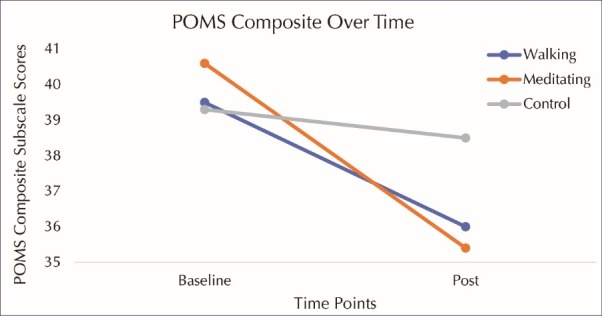
POMS composite over time.

Table 2 displays the mood state scores at various time points throughout the visit. There were no significant group x time interaction effects for the depression/dejection (*P* = 0.20, η^2^= 0.05) or anger/hostility (*P* = 0.18, η^2^= 0.05) subscales. As displayed in Figures 1 and 2 (respectively), a significant group x time interaction effect emerged when evaluating the fatigue/inertia (*P* = 0.04, η^2^= 0.10) subscale as well as the POMS composite scores (*P* = 0.05, η^2^= 0.09). Post hoc paired *t* tests revealed that fatigue/inertia subscale scores significantly decreased from baseline to post-intervention in both the exercise (*P* = 0.03) and meditation (*P* < 0.001) groups. Post-intervention scores were significantly lower in the 2 intervention groups compared to the control group (*P* < 0.05). However, POMS composite scores decreased significantly in the meditation group (*P* < 0.001) but not in the exercise group (*P* = 0.10).

## Discussion

Both exercise and meditation have been shown to have psychological benefits,^9-12^ yet few controlled experimental studies have elucidated how single bouts of these 2 activities compare when considering psychological outcomes. The aim of the current study was to directly compare the effects of 10-minute sessions of brisk walking and meditation on mood profile among a young adult sample. We hypothesized that both walking and meditating, compared to an inactive control group, would result in mood improvements. Though both exercise and meditation induced some level of mood enhancement, only meditation resulted in a statistically significant improvement of overall mood profile. When evaluating the 3 subscales of mood profile (as identified by the POMS questionnaire), both exercise and meditation resulted in statistically significant reductions in fatigue/inertia. No significant changes were observed for depression/dejection or anger/hostility subscales.

Our findings are in alignment with previous studies demonstrating mood benefits as a result of mindfulness meditation. For example, Jain and colleagues^32^ evaluated the effects of short-term (i.e., 1-month) mindfulness meditation training, concluding that mindfulness meditation decreased distress levels and increased positive mood. In support of our observed reductions in fatigue/inertia, a study by Tang et al^33^ found five 20-minute sessions of integrative meditation to significantly reduce anxiety, depression, anger, and fatigue while increasing vigor. In contrast to the aforementioned studies, however, the present study evaluated a single bout of meditation.

Single, short-duration (e.g., 5-minute) bouts of mindfulness have previously been shown to enhance perceived state mindfulness.^34^ Results from our manipulation check indicate that participants felt they had engaged in mindfulness. As such, we feel comfortable considering mindfulness-related mechanisms to explain the observed findings. For example, a core aspect of mindfulness is the promotion of self-awareness.^23^ The mindfulness meditation script used within the present study incorporated a brief body-scan, prompting participants to identify any areas of the body that felt particularly tense, and encouraging participants to relax into those areas. It is possible that this process of relaxing and releasing tension resulted in lower levels of subjective post-meditation fatigue.

There is also a neural basis of biological plausibility for the observed findings. A review by Tang and colleagues^35^ suggested that mindfulness meditation (mainly) affects the prefrontal cortex, insula, striatum, and amygdala. The authors conclude that mindfulness meditation may improve one’s well-being via neural circuitry involved in the regulation of emotions, self-awareness, and present-moment awareness. The aforementioned areas of the brain are also important for mood regulation. To illustrate, neuroimaging research has revealed abnormalities in the prefrontal cortex and amygdala among persons with depression, with some of the abnormalities being dependent upon the individuals’ mood state.^36^

Our findings suggest that, along with meditation, exercise may also improve fatigue-related mood state. This may be due, in part, to increased levels of arousal. Participants’ awareness of increased physiological arousal (e.g., elevated heart rate) in addition to general awareness of the act of ambulating at a brisk pace (as opposed to sitting) may have resulted in participants’ perceiving lower levels of lethargy and other fatigue-related parameters. However, the question remains, “Why did exercise not result in significant improvements in overall mood, whereas meditation did?” It is possible that a 10-minute brisk walk was not a distinct enough stimulus from participants’ typical ambulation activities and walking pace. Our sample population, which on average exceeded double the recommended amount of weekly moderate-to-vigorous physical activity, perhaps did not find this bout of exercise to be mood enhancing given it’s relatively low-intensity (compared to moderate or vigorous activities that they routinely engage in). We do not believe that the walking duration was too short to elicit positive mood changes, as our previous work^37^ has demonstrated the capability of a single 5-minute bout of brief walking to significantly improve mood state in a similar population of young adults. Of note, differential neural activation patterns may also help explain why meditation and walking did not result in the same mood effects. For example, the motor cortex has been evidenced to play an important role in activating muscles involved in ambulation (e.g., dorsiflexors and plantarflexors).^38^ There would be no need for this level of activation during a seated or lying meditation. Relatedly, Dietrich’s transient hypofrontality hypothesis^39^ suggests that submaximal exercise may result in the down-regulation of neural activity not directly involved in motor control, such as the prefrontal cortex (which is thought to be activated during meditation, especially among novices).

The present study is not without limitations. One limitation is the utilization of a relatively small sample size of 22 participants/group. However, it is unlikely that the study was under-powered, given the ability to detect significant group x time interactions. An additional limitation of the study is that our sample was made up of young, highly-active college-aged students, so our findings should be interpreted cautiously when considering other populations. However, as mentioned previously, this is a notable population to study within this context as depressed mood has been shown to associate with worse academic performance among college students, and a significant proportion of college student are influenced by mental health concerns (e.g., approximately 15% of undergraduate students have some form of diagnosed anxiety or depression).^40^ On the contrary, the intervention protocols (i.e., 10-minute brisk walk or guided mindfulness meditation) are widely generalizable; they are accessible to anyone who possesses the physical ability to walk, access to a walking location (e.g., treadmill, track, sidewalks), and access to a guided meditation (e.g., meditation application for a phone, YouTube video, DVD). Another limitation to the current study is that we were not able to delineate mechanisms of the observed effects. Notably, however, this was not the aim of the study. Further, we did not evaluate the test-retest reliability of the POMS assessment in our sample, which is a limitation of our study. Despite these limitations, the current study has a number of strengths that demonstrate its important contribution to the literature surrounding exercise and meditation psychology. Main strengths of the current study include its experimental, controlled design and its novelty. This is the first study, to our knowledge, to directly compare a single 10-minute bout of exercise and a single 10-minute bout of meditation to examine the effects on mood profile. There is a need for more comparison studies on single doses of these activities.

Future work comparing acute aerobic exercise and mindfulness meditation may consider replicating the current study design. Though it is important to conduct follow-up studies to extend research findings, replication will always be a critical step in the process of hoping to understand relationships. This study should also be replicated using a within-subjects design. Future acute exercise/meditation comparisons studies may also consider adding additional groups (e.g., yoga or mindful walking) to help tease out which components of meditation/walking may be eliciting mood alterations. Additionally, studies involving acute meditation should be sure to measure whether or not participants have previous meditation experience, or may wish to take this factor into consideration as a potential exclusionary criterion (depending on the specific research question).

We live in a society that seems to glorify having a busy schedule. Most adults are not regularly active enough,^41^ with a commonly reported reasoning for this being a “lack of time.”^42^ Notably, affect during exercise has also been implicated in the formation of future intentions/behaviors related to exercise.^43^ The implemented protocol serves as a feasible solution to both of the aforementioned factors. It should be fairly reasonable to incorporate a 10-minute walk or meditation into one’s schedule. Additionally, participants in the present study rated the exercise to be enjoyable, which makes it plausible that they had positively valenced affective experiences during the walking bout.

## Conclusion

In conclusion, the current study evaluated the effects of a 10-minute bout of brisk walking and meditation (in comparison with sitting) on mood profile among active young adults. Meditation was found to improve overall mood state, as well as a fatigue/inertia subscale of mood profile, whereas walking was found to only improve the fatigue/inertia subscale of mood. Thus, individuals wanting overall mood enhancement may find a 10-minute meditation to be more effective than walking or sitting for 10 minutes. Individuals wanting to more specifically decrease fatigue-related mood state may consider either a brisk walk or a guided meditation. Though both 10-minute bouts of brisk walking and meditation demonstrate some potential to induce mood state benefits, future work is needed to confirm these findings and contribute additional research to the acute exercise/meditation comparison literature.

## Ethical approval

All procedures performed in studies involving human participants were in accordance with the ethical standards of the institutional and/or national research committee and with the 1964 Helsinki declaration and its later amendments or comparable ethical standards. This study was approved by the authors’ institutional review board.

## Informed Consent

Informed consent was obtained from all individual participants included in the study.

## Competing interests

The authors declare no conflicts of interest.

## Authors’ contributions

ME collected the data. ME performed analyses and drafted the manuscript. All authors have reviewed and provided intellectual feedback on the manuscript. All authors have read and approved the final version of the manuscript and agree with the order of presentation of the authors.

**Table 1:** Demographic characteristics of the analyzed sample (N = 66)

**Variable**	**Group**			
	**Walking (n = 22)**	**Meditating (n = 22)**	**Control (n = 22)**	***P*** **value** ^a^
Age, mean	21.5 (2.3)	22.6 (3.2)	20.9 (2.5)	0.12
Gender, % male	27.3	22.7	36.4	0.60
Race, %				0.49
Mexican American	0.0	4.5	4.5	
Other Hispanic	4.5	0.0	0.0	
Non-Hispanic White	54.5	72.7	77.3	
Non-Hispanic Black	31.8	13.6	9.1	
Other	9.1	9.1	9.1	
BMI, mean, kg/m^2^	25.3 (5.4)	24.3 (4.4)	24.6 (4.5)	0.53
MVPA, mean, min/week	378.1 (414.2)	294.4 (247.1)	328.5 (416.3)	0.76
Last meal, mean, h	5.2 (4.8)	4.6 (4.5)	4.0 (3.7)	
Previous meditation experience, %	36.4	59.1	59.1	0.21
Currently meditating, %	50.0	50.0	50.0	1.0
Exercise enjoyment, mean	4.3 (.8)	4.3 (.8)	4.5 (.6)	0.67
Stress responses, mean	70.9 (10.9)	67.0 (12.6)	66.0 (11.2)	0.36
Mindfulness, mean	42.4 (7.4)	40.3 (7.0)	39.5 (7.2)	0.41
Emotion regulation, mean	67.6 (15.1)	73.5 (21.1)	72.6 (19.8)	0.56
Dysexecutive function, mean	45.3 (10.4)	43.1 (10.0)	47.8 (14.9)	0.44
Heart Rate, mean				
Resting	71.7 (11.0)	75.8 (10.9)	67.9 (12.6)	
Mid-intervention	113.10 (21.3)	65.2 (13.5)	69.2 (14.5)	
End-intervention	93.55 (16.0)	64.1 (14.3)	68.9 (14.8)	
Final heart rate	76.2 (15.3)	74.6 (13.8)	67.9 (14.1)	
RPE, mean	9.5 (1.7)	N/A	N/A	N/A
Speed, mean, mph	3.2 (.4)	N/A	N/A	N/A

All values presented as mean (standard deviation). ^a^ *P* values calculated via ANOVA tests of between-subject effects (continuous data) and via chi square tests (categorical data). BMI = body mass index; Currently meditating = proportion of the group who reported having previous meditation experience who were practicing meditation at the time; Dysexecutive function = composite score from the Dyxexecutive Questionnaire (DEX); Emotion regulation = composite score from the Difficulties in Emotion Regulation Scale (DERS); Last meal = time elapsed since last consuming food; Mindfulness = composite score from the Freiburg Mindfulness Inventory (FMI); MVPA = moderate-to-vigorous physical activity; N/A = not applicable; Previous meditation experience = proportion of the group who had any previous experience with meditation; Resting= resting heart rate; RPE = rating of perceived exertion using standard Borg 6-20 rating scale; Speed = selected miles per hour to walk at during treadmill walking bout; Stress responses = composite score from the Responses to Stressful Experiences Scale (RSES).

**Table 2:** POMS Scores

**Variable**	**Group**			
	**Walking**	**Meditating**	**Control**	***P*** **value** ^a^
Mood, mean score				
Depression/dejection				0.20
Baseline	14.5 (2.7)	15.1 (3.8)	14.1 (2.1)	
Post	14.2 (3.4)	13.9 (1.9)	14.0 (2.0)	
Anger/hostility				0.18
Baseline	12.1 (2.2)	12.4 (2.0)	12.1 (2.1)	
Post	11.7 (1.3)	11.4 (1.1)	12.0 (1.5)	
Fatigue/inertia				0.04
Baseline	12.9 (5.0)	13.1 (5.9)	13.1 (4.8)	
Post	10.1 (5.0)^b^	10.1 (3.4)^c^	12.5 (4.8)	
Composite				0.05
Baseline	39.5 (8.7)	40.6 (10.2)	39.3 (7.3)	
Post	36.0 (9.0)	35.4 (5.6)^c^	38.5 (7.2)	

All values presented as mean (standard deviation). ^a^ *P* values calculated via 3 x 2 repeated measures ANOVA tests: between-subjects factor = group, within-subjects factor = time. ^b^ Post hoc paired *t* test (baseline vs. post) *P* < 0.05. ^c^ Post hoc paired *t* test (baseline vs. post) *P* < 0.001.
